# Intraventricular Anaplastic Pleomorphic Xanthoastrocytoma: Very Rare Localization and Early Recurrence of a Rare Tumor

**DOI:** 10.7759/cureus.2665

**Published:** 2018-05-21

**Authors:** Fabio Roberti, Martin Baggenstos

**Affiliations:** 1 Neurosurgery, Indian River Medical Center; 2 Neurological Surgery, George Washington University, Washington, USA

**Keywords:** intraventricular tumor, pleomorphic xanthoastrocytoma, anaplastic glioma

## Abstract

We report a rare case of an adult, with no previous history of seizures, found to have a large intraventricular Anaplastic Pleomorphic Xanthoastrocytoma (APXA). To the best of our knowledge, this is only the second documented report of an APXA located within the ventricular system in an adult. The tumor was characterized by anaplastic features and necrosis without an elevated mitotic index, and it recurred shortly after a gross total surgical resection.

## Introduction

Pleomorphic xanthoastrocytomas (PXA) are rare low-grade astrocytic tumors that account for less than 1% of all brain neoplasms. Frequently cystic with a mural nodule [1-2] and usually located to involve the temporal lobe cortex and leptomeninges [1,3], these tumors are predominately found in children and young adults. Clinically, they often manifest with partial or generalized seizures secondary to their predominant superficial location [3-4], especially in the temporal lobe. First described by Kepes and colleagues in 1979, PXA was added to the World Health Organization (WHO) classification of tumors of the central nervous system (CNS) in 1993. Although PXAs have been given a classification of a WHO grade II tumor, 9%-20% of such tumors have been reported to show increased mitotic activity (defined as five or more mitoses in 10 high-power fields) and/or areas of necrosis on histologic evaluation [5]. In 2007, the WHO classification of CNS tumors recognized this subset of lesions with aggressive histologic features and clinical behavior as “PXA with anaplastic features” and, more recently, the 2016 classification saw the addition of anaplastic pleomorphic xanthoastrocytomas (APXA) as a new entity to label these rare tumors. Anaplastic PXA shares some pathological characteristics with glioblastomas [5-6], making such a differential diagnosis, at times, challenging. We report a rare case of an intraventricular anaplastic pleomorphic xanthoastrocytoma, which recurred a few months after a gross total surgical resection.

## Case presentation

Preoperative evaluation

**Figure 1:**
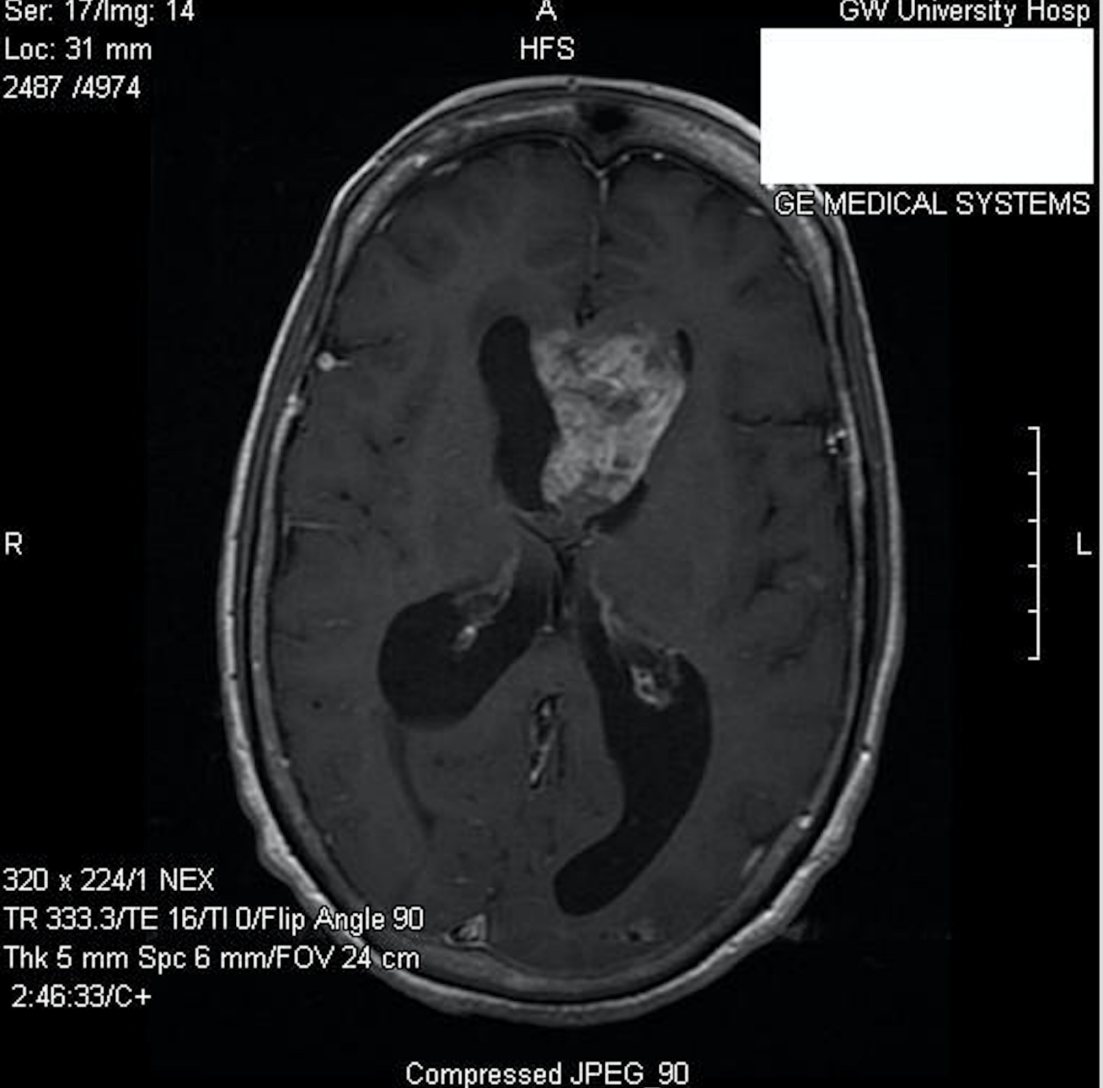
Preoperative axial post-contrast T1-weighted MR image MR: magnetic resonance

**Figure 2:**
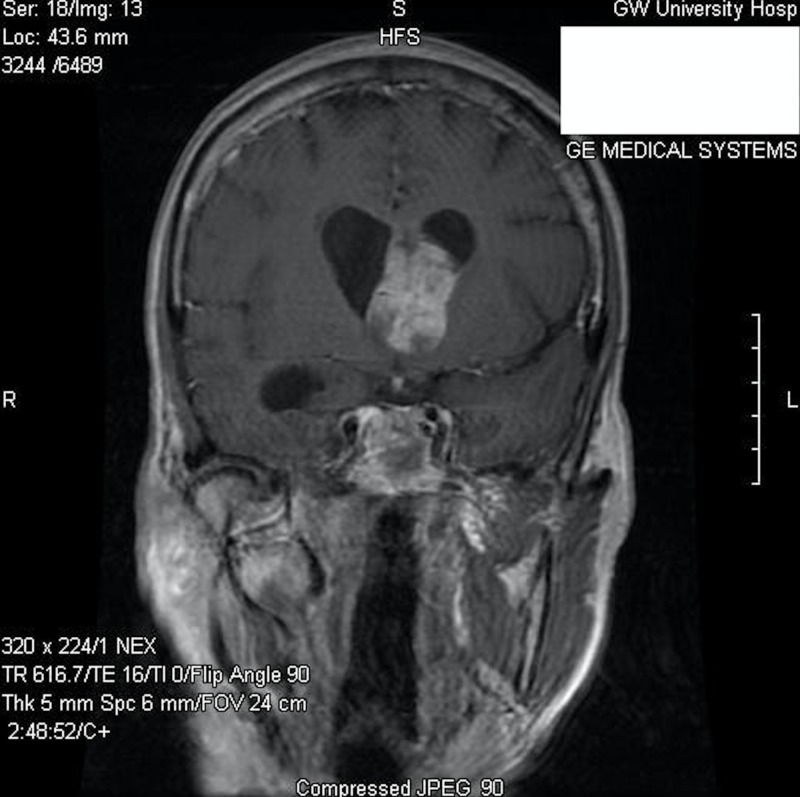
Preoperative coronal post-contrast T1-weighted MR image MR: magnetic resonance

A 65-year-old female, who had been previously healthy, presented to the emergency department at our institution with a six-month history of memory impairment, urinary incontinence, and ataxia. On physical examination, she was alert and oriented, but with difficulty remembering recent and past events. Her cranial nerves were intact, and she demonstrated a normal motor and sensory examination. She had no history or clinical findings of tuberous sclerosis. Contrasted magnetic resonance imaging (MRI) of the brain demonstrated a 4.9 X 3.0 cm heterogeneously enhancing intraventricular mass, centered on the septum pellucidum and extending into the lateral ventricles, with associated obstructive hydrocephalus (Figures 1-2).

Operation and postoperative course

**Figure 3:**
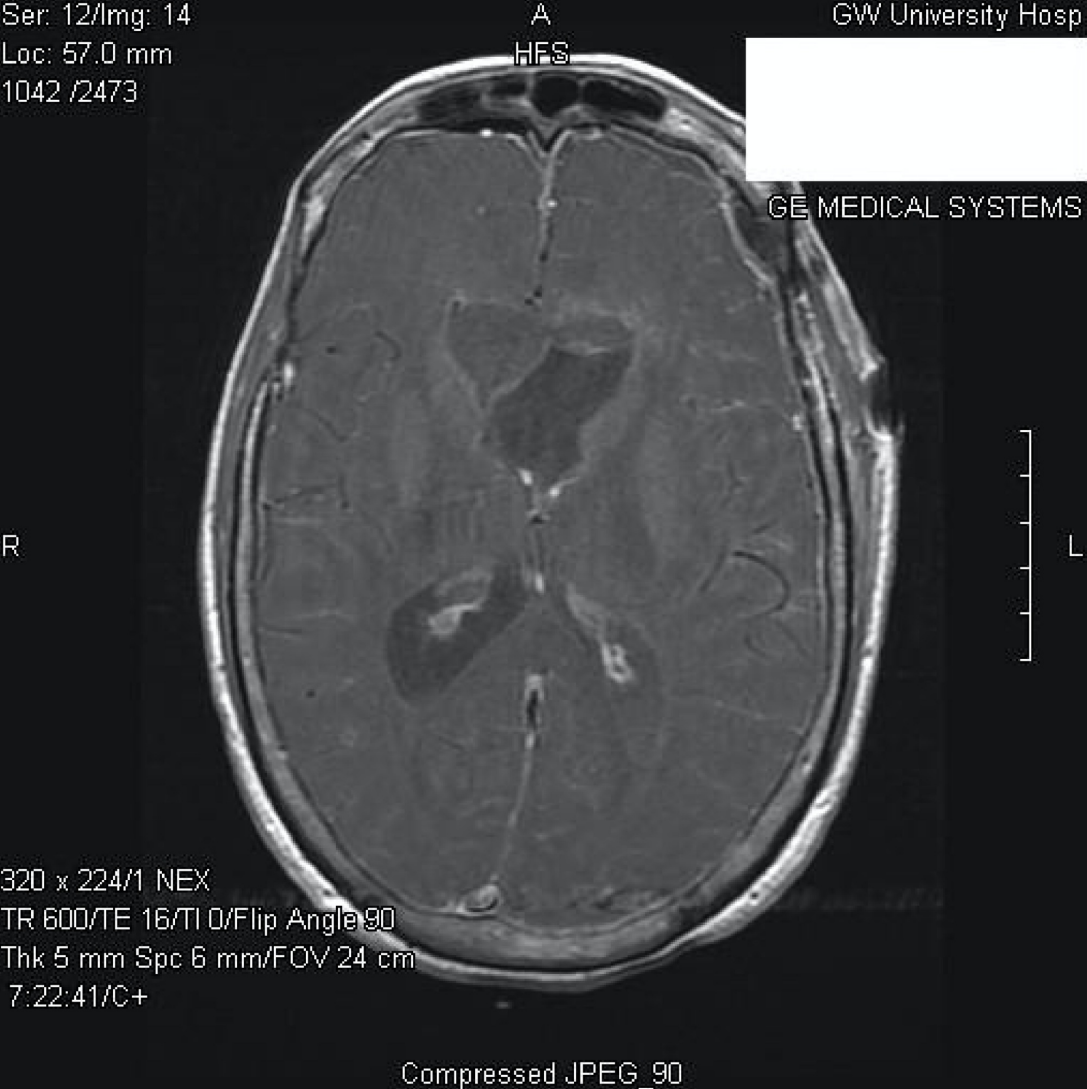
Postoperative axial post-contrast T1-weighted MR image MR: magnetic resonance

**Figure 4:**
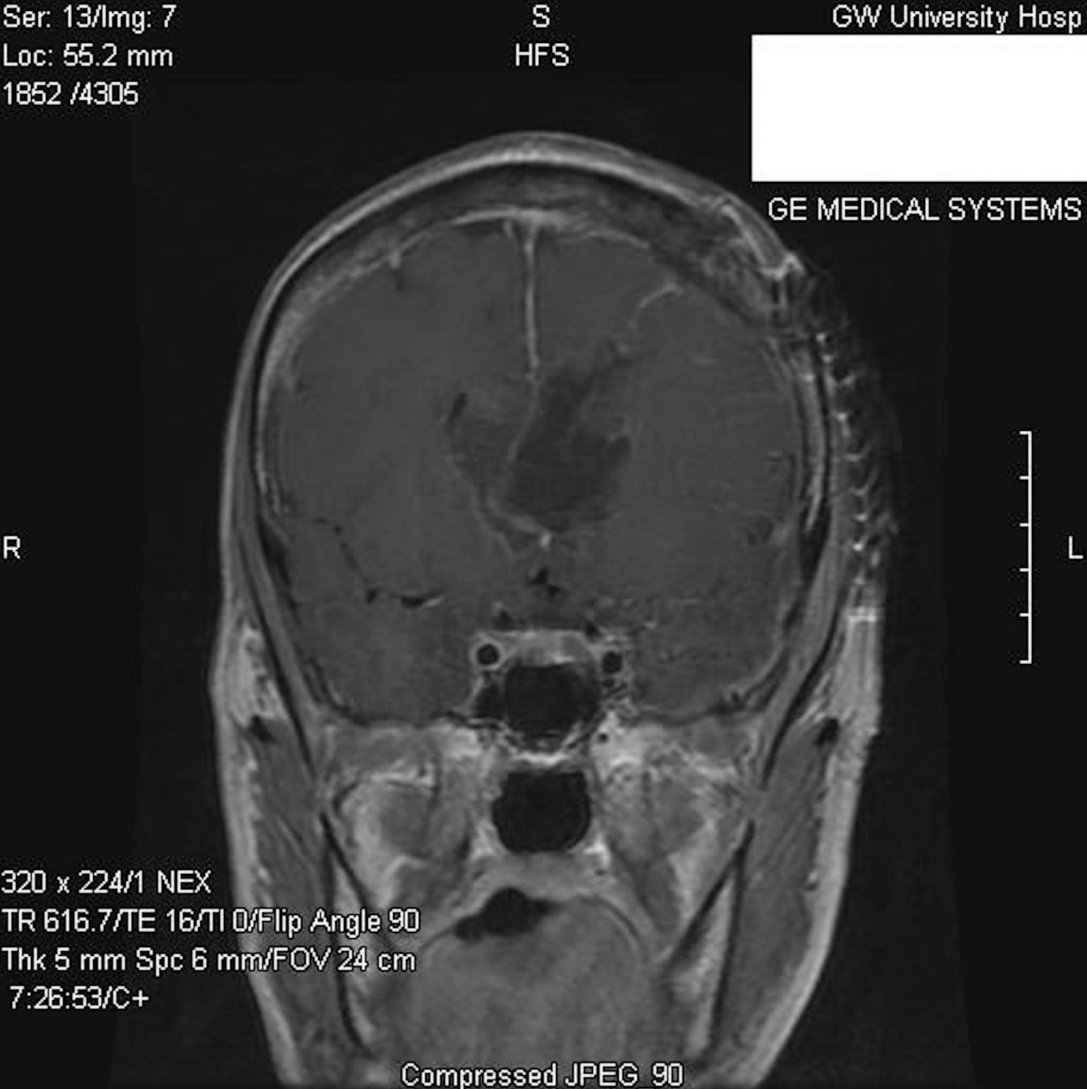
Postoperative coronal post-contrast T1-weighted MR image MR: magnetic resonance

**Figure 5:**
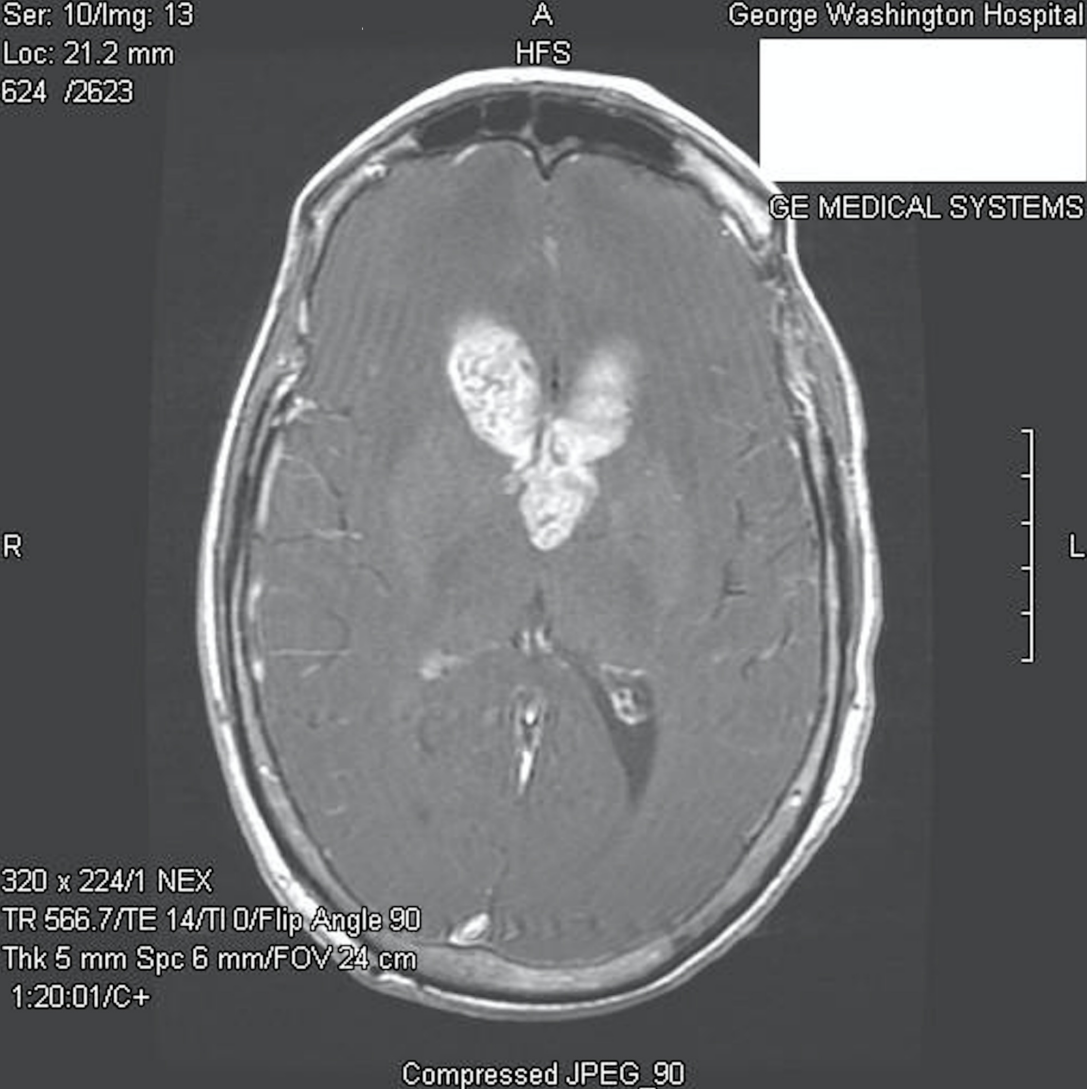
Tumor recurrence three months after resection

The patient was then taken electively to the operating room for the resection of the intraventricular ventricular mass via a left frontal craniotomy, with a corticectomy through the middle frontal gyrus. Once the ependymal layer of the left lateral ventricle was opened, a grayish slightly vascularized mass was found. An interface between the ventricle and the tumor was developed and a dissection plane was created between the anterior portion of the tumor, which was located underneath the corpus callosum, and the medial component centered on the septum pellucidum. Postoperatively, the patient experienced a transient mutism, which began to resolve a few weeks after the operation. A gross total resection was achieved (Figures 3-4) and a ventriculoperitoneal shunt was placed due to the presence of a continued postoperative hydrocephalus. Upon confirmation of the pathological diagnosis of anaplastic pleomorphic xanthoastrocytomas (at the time of the surgery, pleomorphic xanthoastrocytoma “with anaplastic features”), adjuvant radiotherapy was considered but, in light of the transient mutism, postponed. Three months later, the patient experienced an episode of confusion with worsening gait instability. Repeated imaging revealed the recurrence of the tumor now involving the lateral and third ventricles (Figure 5). Due to the extent of the disease, the patient’s clinical condition and her wishes against pursuing the further invasive or adjuvant treatments, conservative and supportive management only were followed.

Histopathological and immunohistochemical examination

**Figure 6:**
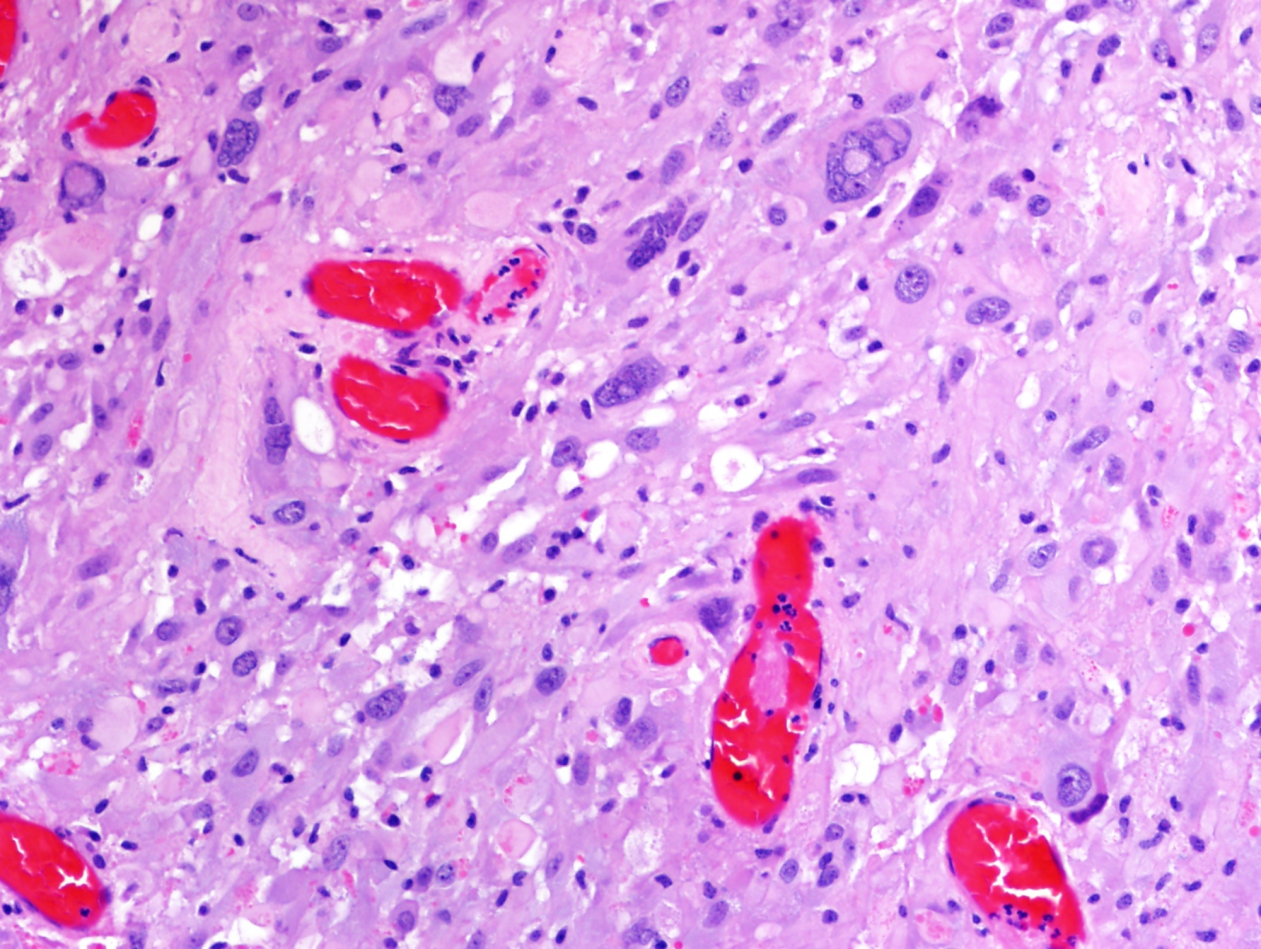
Formalin-fixed paraffin-embedded H & E sections Hematoxylin and eosin (H & E) section demonstrating neoplastic cells with enlarged multilobed or bizarrely shaped nuclei and frequent cytoplasmic pseudoinclusions

**Figure 7:**
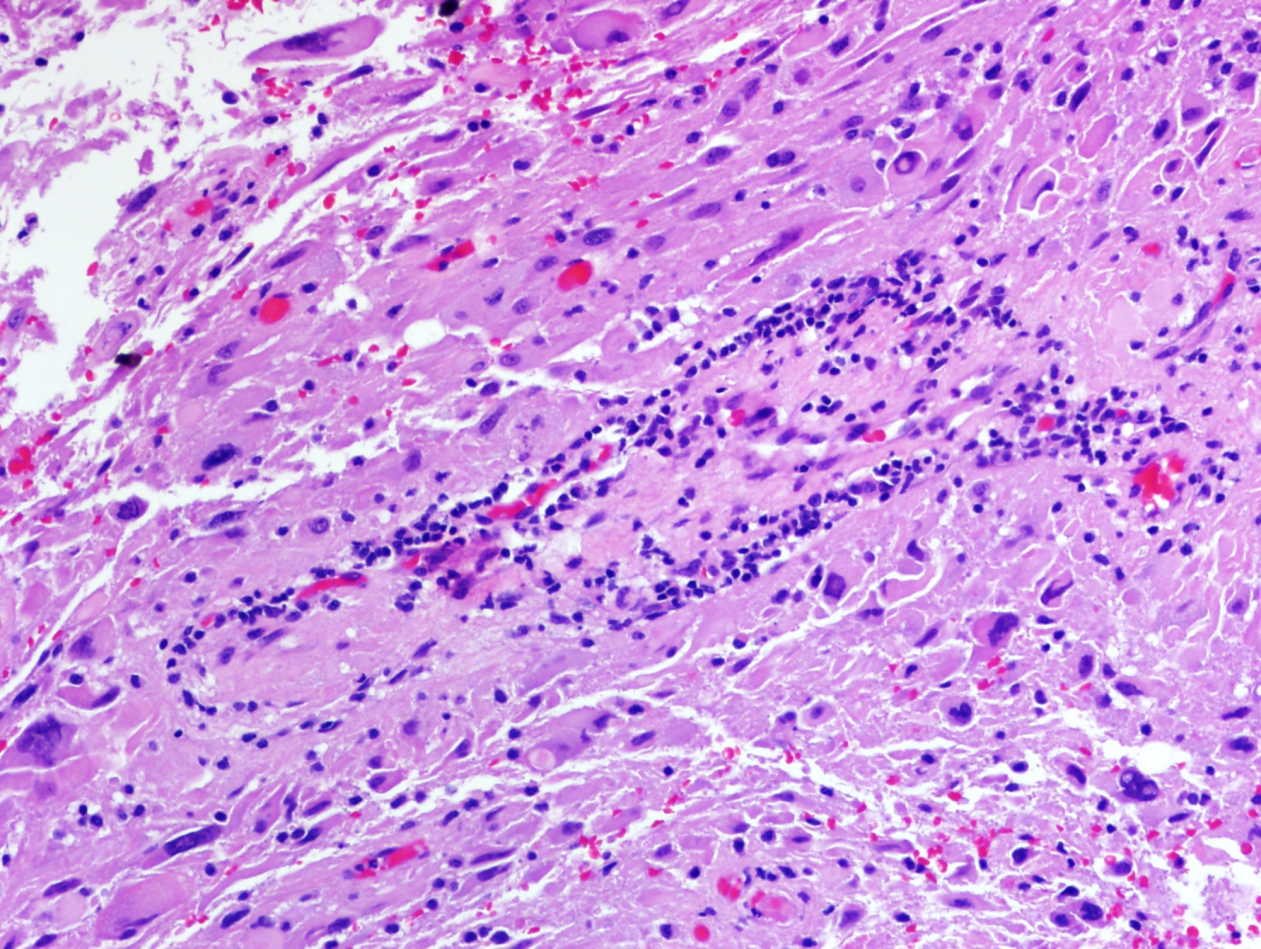
Lymphocytes scattered among tumor cells and around blood vessels

**Figure 8:**
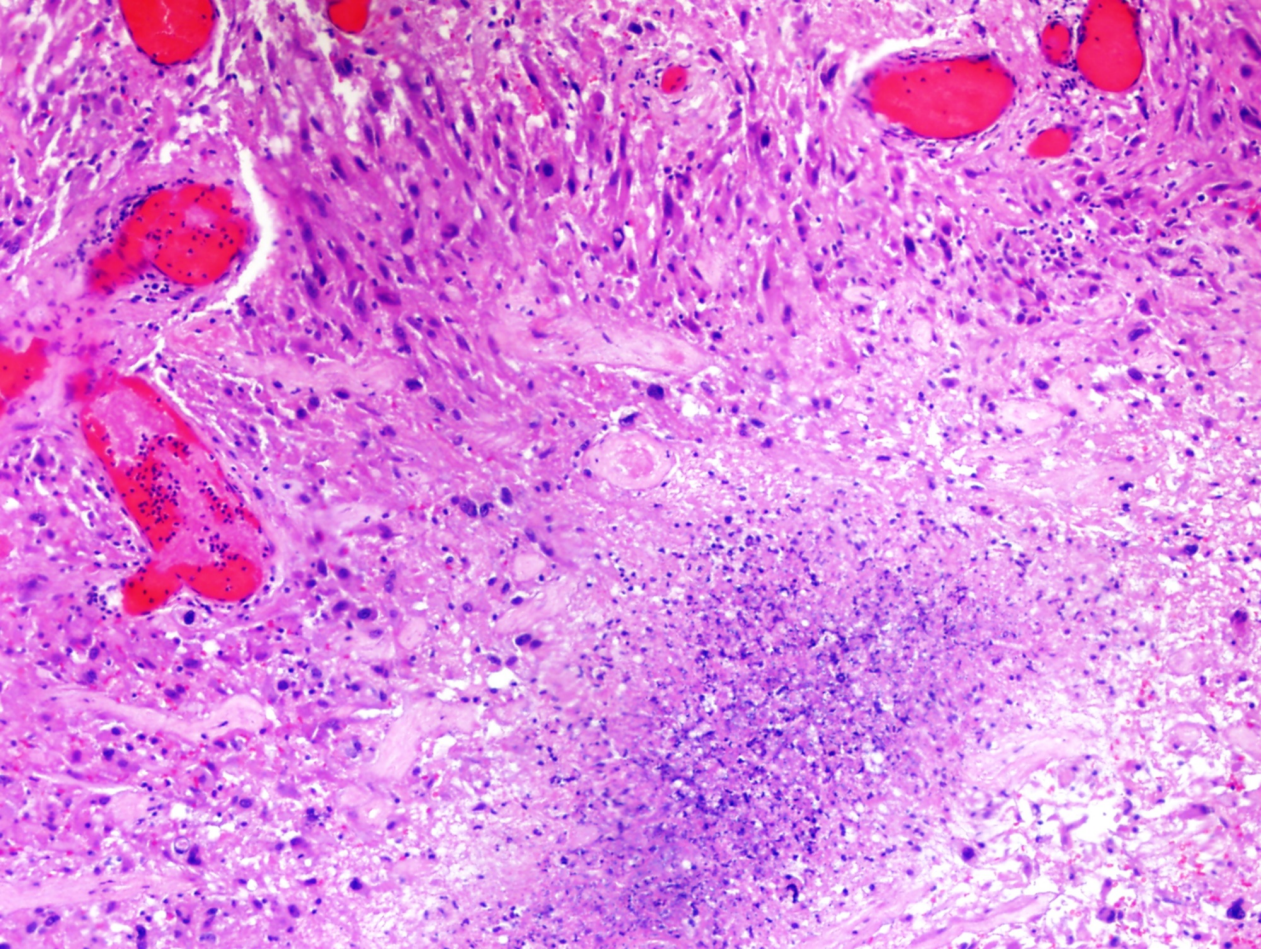
Necrotic zones surrounded by the pseudopalisaded appearance of spindled tumor cells

**Figure 9:**
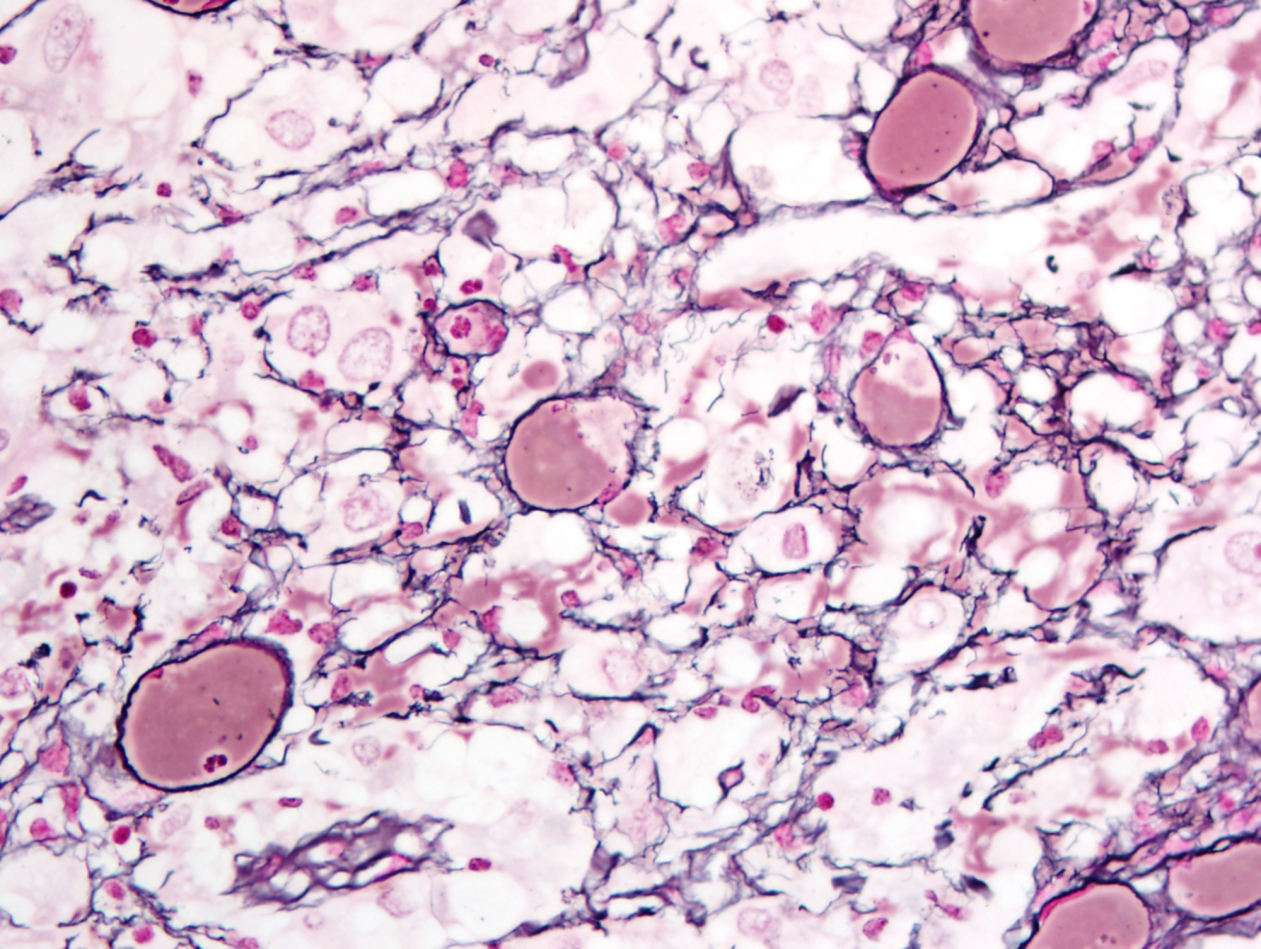
Focal reticulin investment of individual tumor cells and small tumor nests

**Figure 10:**
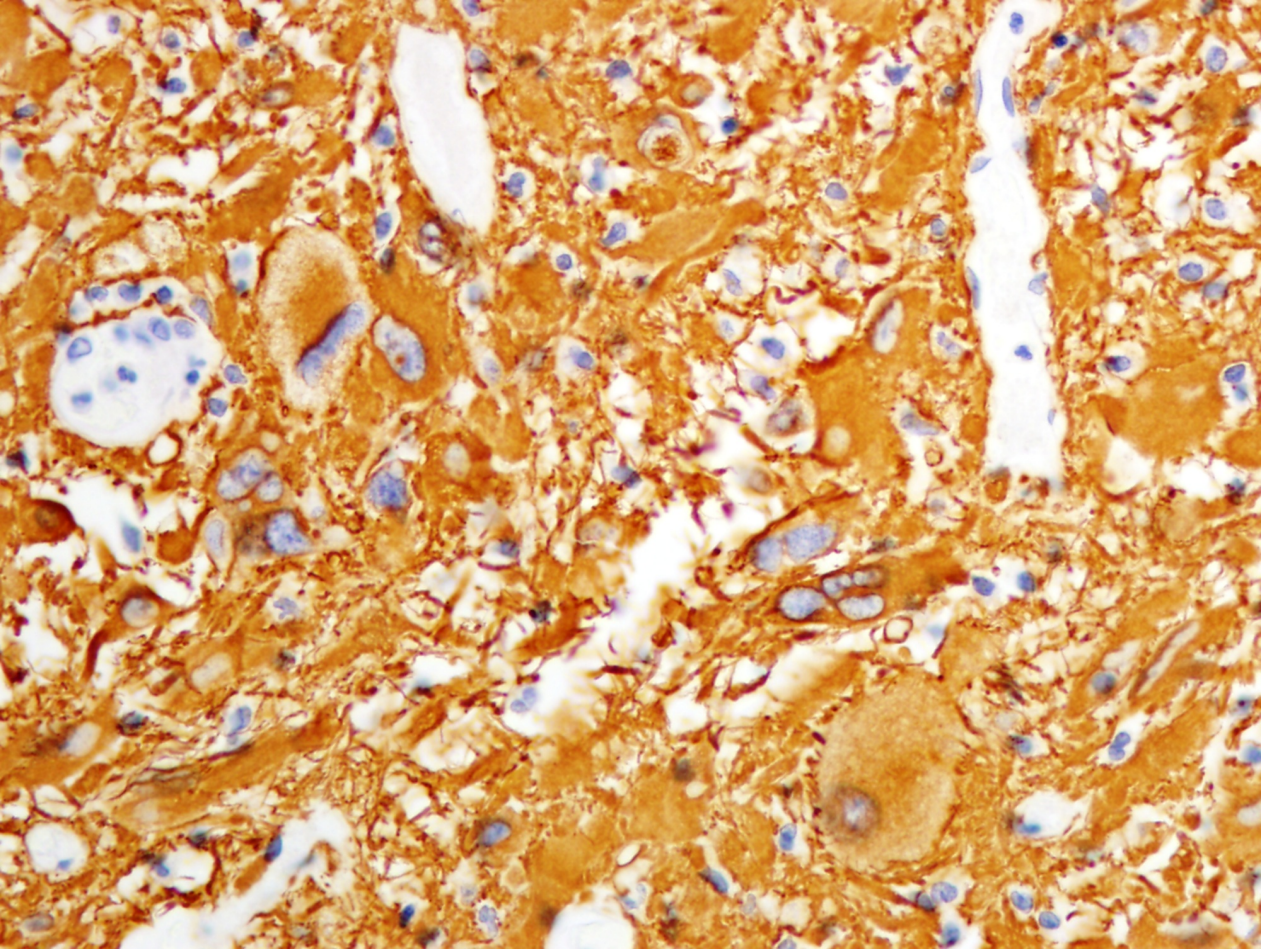
Immunohistochemical staining with tumor cells positive for GFAP GFAP: glial fibrillary acidic protein

**Figure 11:**
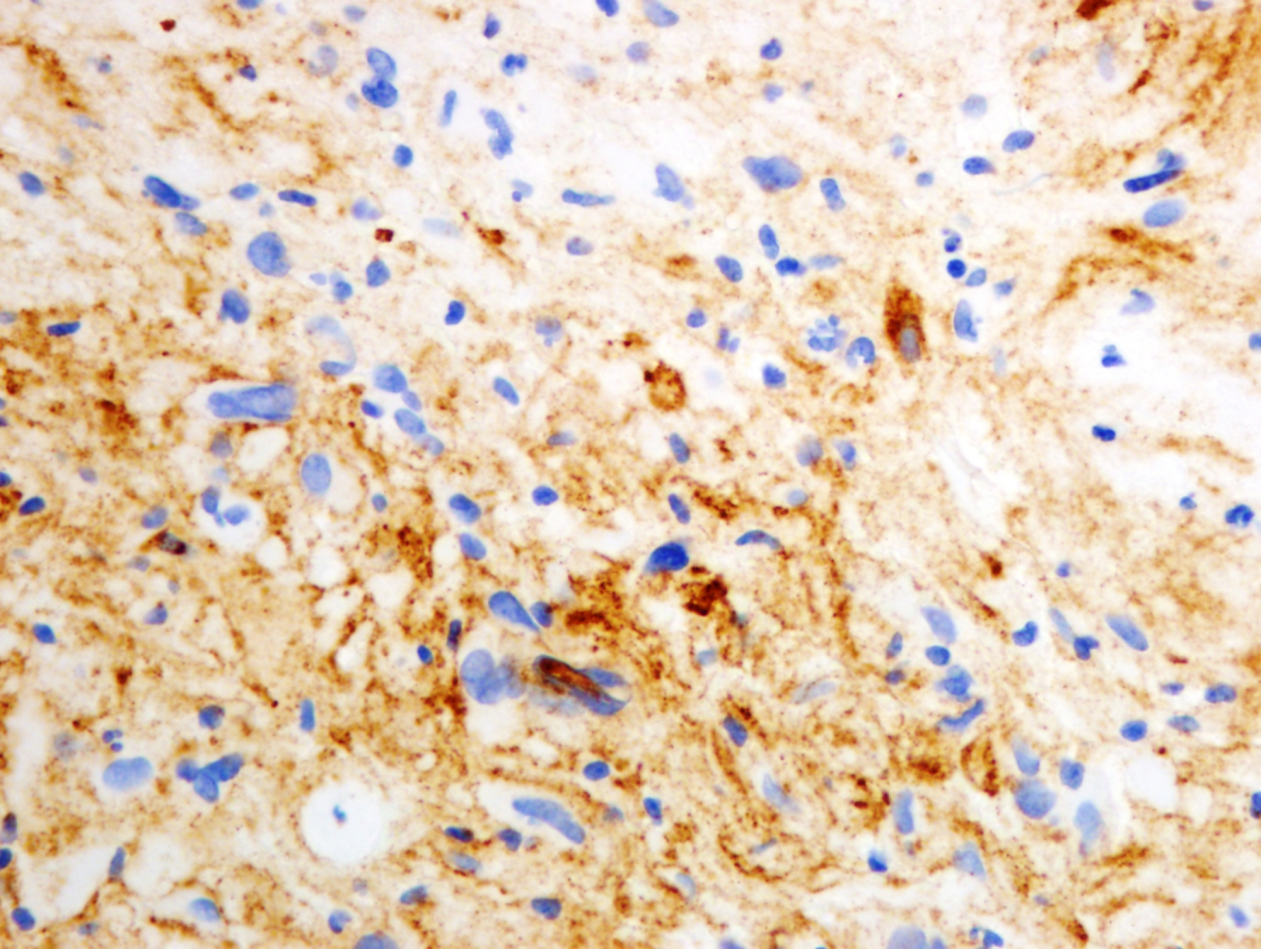
Immunohistochemical staining with tumor cells positive for CD34

**Figure 12:**
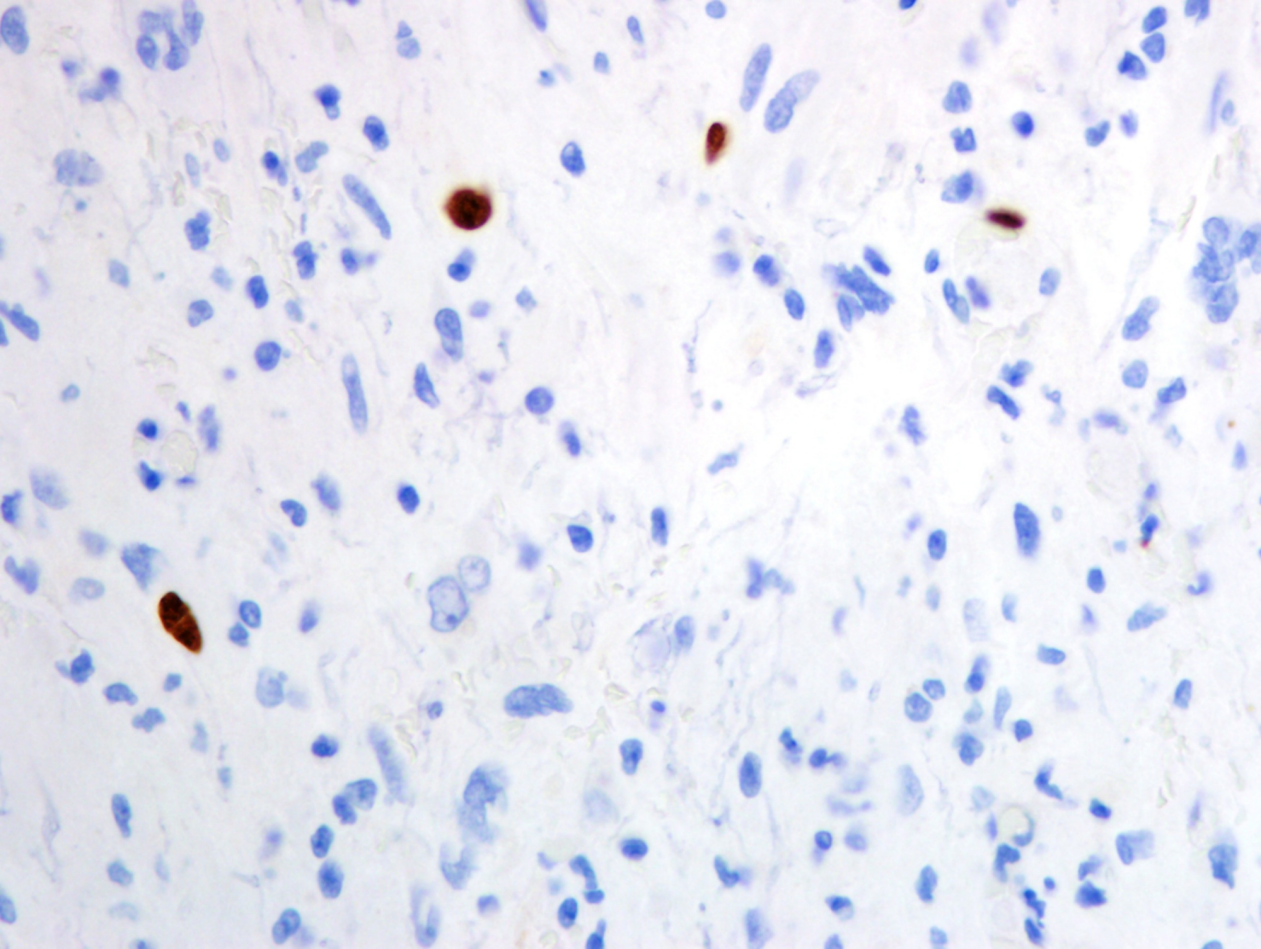
Proliferation index of 2% with Ki-67

**Figure 13:**
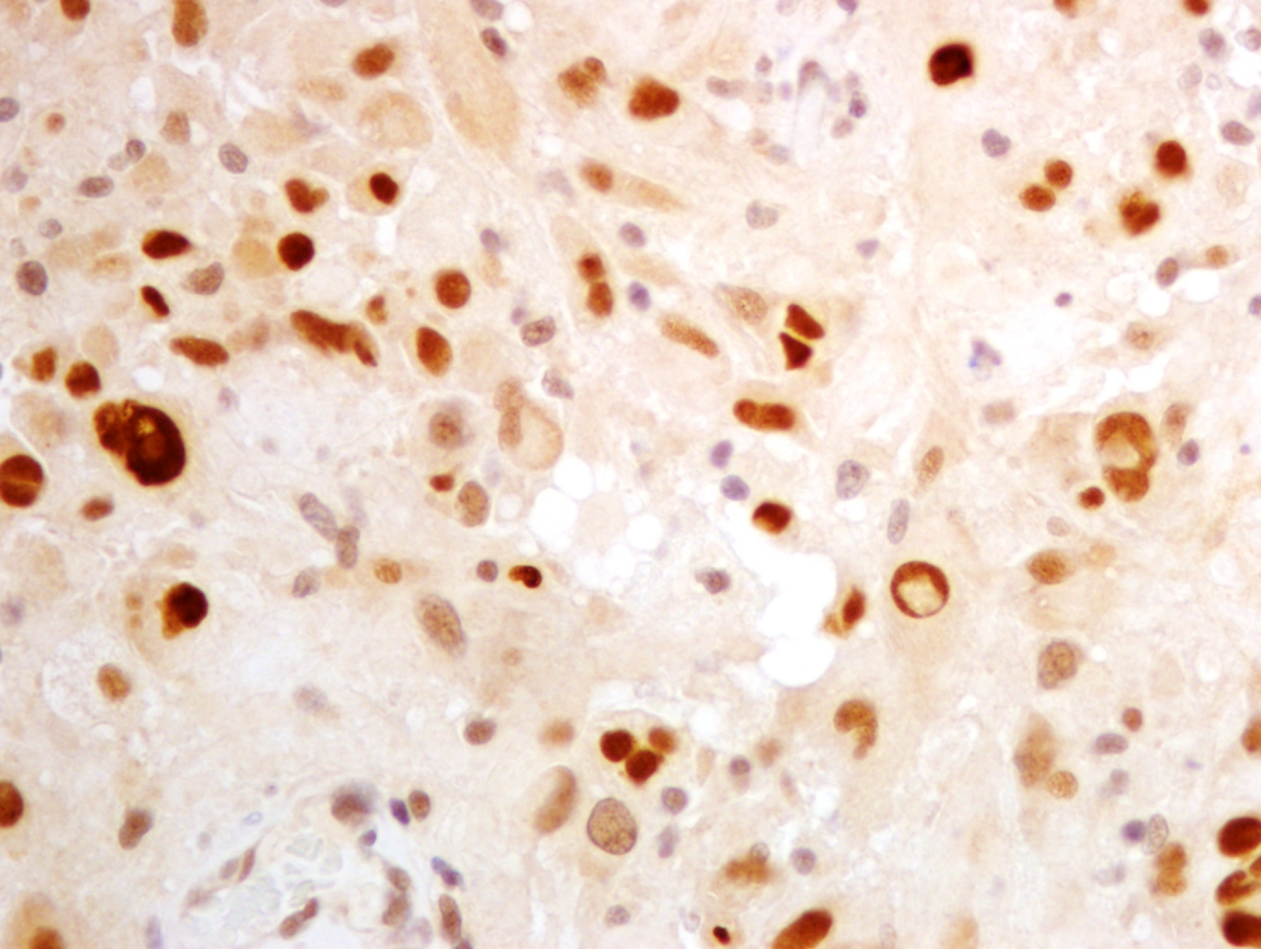
p53 positivity in 33% of neoplastic nuclei

Formalin-fixed paraffin-embedded histologic sections stained with hematoxylin and eosin disclosed a moderately cellular neuropil-forming tumor with cells arranged in fascicles or sheets. Neoplastic cells displayed abundant pink cytoplasm and many had enlarged multilobed or bizarrely-shaped nuclei with frequent cytoplasmic pseudo-inclusions. Xanthomatous change was not conspicuous in neoplastic cells and eosinophilic granular bodies (EGBs) were prominent in the background. Lymphocytes were scattered among tumor cells and around blood vessels. Mitoses were absent. Blood vessels showed prominent congestion without cellular proliferation or glomeruloid formations. There were several foci of necrosis characterized by smudged granular pink debris containing karyorrhectic fragments. A few of these necrotic zones were surrounded by radially oriented spindled tumor cells, imparting a pseudopalisaded appearance. Reticulin stain showed the focal reticulin investment of individual tumor cells and small tumor nests (Figures 6-9). Calcification was not detected microscopically. Immuno-histochemical staining demonstrated that tumor cells were strongly and diffusely positive for glial fibrillary acidic protein (GFAP) and focally positive for CD34. Tumor cells did not mark with a synaptophysin immunostain. Ki-67 immunostain yielded a proliferation index of 2%. Immunostain for p53 disclosed positivity in 33% of neoplastic nuclei (Figures 10-13).

Nuclear pleomorphism, abundant EGBs, the absence of mitoses and vascular proliferation, reticulin investment of tumor cells, and GFAP and CD34 immunopositivity with a low proliferation index were morphologic features leading to the classification of this tumor as PXA. The recognition of the microscopic foci of necrosis resulted in a final diagnosis of APXA, a diagnosis to which the WHO grading system for CNS tumors now assigns a grade III.

In an adult astrocytic tumor with exaggerated nuclear pleomorphism, one is more likely to see tumor necrosis and significant p53 immunopositivity in giant cell glioblastoma than in PXA. The investment of tumor cells by reticulin and perivascular lymphocytic infiltrates have also been observed in giant cell glioblastoma. The absence of mitoses and vascular proliferation and the abundance of EGBs caused us to favor a variant of PXA rather than a glioblastoma subtype.

## Discussion

PXAs have been originally classified as rare, low-grade (WHO grade II) astrocytic tumors of young adults. Although most typically located in the cerebral hemispheres, PXAs have been reported to rarely involve other sites, such as the hypothalamus, spinal cord, sella, cerebellum, and retina [2,5-12]. PXAs are usually found in a superficial location and are generally associated with a good prognosis after complete surgical resection [1,13]. To our knowledge, only a single PXA with anaplastic features (now APXA) has been previously reported to involve the ventricular system (lateral and third ventricles) [14].

Microscopically, PXAs are characterized by the presence of prominent pleomorphism, variably conspicuous xanthomatous tumor cell change, EGBs, focal reticulin elaboration, and perivascular lymphoid infiltration. The presence of tumors with anaplastic features, defined as five or more mitoses in 10 high-power fields and/or necrosis, as well as the potential for a secondary anaplastic transformation have been reported in the literature [3]. In 2007, the revised WHO classification of tumors of the CNS suggested the designation “PXA with anaplastic features” to describe this particular subset of PXAs with five or more mitoses per 10 high-power fields and/or areas of necrosis [15]. PXAs with increased mitoses, vascular proliferation, and/or necrosis can be very difficult to distinguish from anaplastic astrocytomas and glioblastoma, especially the giant cell variant of glioblastoma [4]. Cases of mixed ganglioglioma and PXA have also been described, further complicating the pathological classification of PXA, whose pleomorphism may suggest bizarre ganglionic cells and whose immunoprofile may be positive for neuronal markers [3]. While classic PXA is given a grade of II in the WHO grading system, PXAs with “anaplastic features” are now defined as APXA and assigned WHO grade III. While more aggressive behavior is expected of these tumors as a whole and while many APXAs rapidly recur and/or acquire even more aggressive histologic characteristics, recurrence interval and overall survival differ significantly from case to case [14,16].

In this presented rare case, which, to the best of our knowledge, is only the second documented report of PXA located within the ventricular system, the tumor recurred shortly after gross total surgical resection. It is possible the tumor was already undergoing malignant transformation at the time of the surgery after a long period of indolent and asymptomatic growth in the non-eloquent territory of the lateral ventricle. Such a malignant transformation would lead to rapid recurrence as a fully malignant astrocytic tumor, even after a gross total resection. It has been noted from previous reports that malignant transformation can occur at a rate of 10%-20%, within a period of seven months to 15 years [17-18]. The scenario of early recurrence with the same histologic features as the initial tumor still remains a possibility in this case. Due to a paucity of intraventricular PXAs described in the literature and the lack of long-term follow-up data after complete resection, it is difficult to speculate if the intraventricular location of this patient’s tumor may have played a role in the early recurrence of the lesion, possibly due to a microscopic residual disease involving the ependymal lining.

While in the past, PXAs with “anaplastic features” were not assigned a WHO grade, making the role of adjuvant radiation therapy after a complete or subtotal resection of such tumors unclear and possibly controversial [3], with the new classification, grade adjuvant radiotherapy and chemotherapy have been utilized to achieve longer-term control [19-20]. In our case, we deferred the use of any adjuvant therapy due to the absence of a residual enhancing tumor and the presence of a transient neuropsychological deficit (mutism).

## Conclusions

We report the case of a rare intraventricular APXA (PXA “with anaplastic features” at the time of the diagnosis) that recurred very early after a gross total surgical resection. To the best of our knowledge, this is only the second report in the literature of an APXA involving the ventricular system and the only case presenting as a solid midline lesion. Although a gross total resection was achieved, the tumor recurred very early in the absence of adjuvant therapy. Due to the unique histopathologic findings and the commonly seen aggressive behavior of tumors previously described as PXA with “anaplastic features,” this subset of tumors were newly labeled as APXA and assigned a grade III by the most recent 2016 WHO classification. Surgical resection followed by adjuvant therapies (radiotherapy and possibly chemotherapy) as well as a close postoperative surveillance is warranted in these cases even when a complete resection is achieved. Our report emphasizes that, although very rarely, anaplastic pleomorphic xanthoastrocytomas may present as purely intraventricular lesions and should be considered in the differential diagnosis of contrast-enhancing intraventricular lesions.
